# The Immediate Effects of a Dynamic Orthosis on Gait Patterns in Children With Unilateral Spastic Cerebral Palsy: A Kinematic Analysis

**DOI:** 10.3389/fped.2019.00042

**Published:** 2019-02-21

**Authors:** Elisabete Martins, Rita Cordovil, Raul Oliveira, Joana Pinho, Ana Diniz, Joao R. Vaz

**Affiliations:** ^1^Faculdade de Motricidade Humana, Universidade de Lisboa, Lisbon, Portugal; ^2^Escola Superior de Saúde do Alcoitão, Lisbon, Portugal; ^3^Faculdade de Motricidade Humana, CIPER, Universidade de Lisboa, Lisbon, Portugal; ^4^Universidade Europeia, Lisbon, Portugal; ^5^Department of Biomechanics, University of Nebraska, Omaha, NE, United States

**Keywords:** cerebral palsy, hemiparetic, suit therapy, kinematic gait analysis, dynamic orthosis, physical therapy

## Abstract

This study analyzes the immediate effects of wearing a Therasuit on sagittal plane lower limb angular displacements during gait in children with unilateral spastic cerebral palsy (US-CP). Seven participants (median age = 7.00 years; ranging from 5.83 to 9.00 years) with US-CP, levels I and II of the Gross Motor Function Classification System, were assessed with kinematic gait analysis in three different conditions: (A) Baseline; (B) Therasuit without elastics and (C) Therasuit with elastics. Significant improvements were observed at the hip joint of both lower limbs during most of the gait cycle in participants wearing a Therasuit, including a decrease in the flexion pattern at the initial contact and swing phase in both lower limbs, and an increase in the extension pattern in the paretic lower limb during the stance phase. At the knee joint in the paretic lower limb, significant differences were found between the baseline and Therasuit with elastics conditions on the knee angle at initial contact, and between baseline and both Therasuit conditions on the flexion angle at swing phase. However, the inter-individual variability in kinematic patterns at the knee joint was high. At the ankle joint, decreased plantar flexion at initial contact and increased dorsiflexion during stance and swing phases were observed at the Therasuit with elastics condition, helping to correct the equinus-foot in the paretic lower limb during the whole gait cycle. The Z-values showed large effect sizes particularly for most of the angular hip variables in both lower limbs and for the angular ankle variables in the paretic lower limb. The Therasuit seems to have some positive immediate effects on gait kinematics in children with spastic unilateral cerebral palsy by providing a more functional and safer gait pattern. Future investigations with larger samples are recommended to further support these findings.

## Introduction

Cerebral Palsy (CP) is a group of pediatric disorders presenting movement and posture symptoms caused by disturbances occurring during the development of the fetal or infant brain. Children with unilateral (or hemiplegic) spastic CP (US-CP) have multiple physical impairments, including muscle weakness, sensory loss, and spasticity, in the upper and lower limbs of one side of the body (1), typically on the opposite side of the brain injury (2). Asymmetry between the paretic and the non-paretic sides is common (3), particularly a decrease in muscle volume on the paretic side (4, 5) and lower limb length discrepency (6). US-CP is the most common syndrome in children born at term and is second in frequency only to spastic diplegia among preterm infants (7–9).

Although “*hemiplegia”* refers to disorders affecting only one side of the body, hemiplegic children often also have motor impairments on the non-paretic side, particularly in more severe types of hemiplegia, which are typically characterized by altered gait patterns in both lower limbs (10). Compensatory movement patterns are adopted which often result in functional difficulty and musculoskeletal dysfunction. Physical therapy aims to maximize functional independence and minimize secondary complications (11).

New technologies have been introduced in rehabilitation programs to promote and/or enhance the engagement of children with CP in a variety of physical activities and tasks. For instance, dynamic orthoses (12) address different CP-related problems: balance control (11); limb symmetry, walking speed and cadence (13); trunk control (14); motor function in all categories of Gross Motor Function Measure (15); and self-care (16). Furthermore, since the 1990s, different types of therapeutic suits have been used in children with CP (16–18), such as Theratogs (TTs) and Therasuit® (TS). TTs are custom manufactured lycra garments covering the trunk and limbs that exert a compressive force on the body (16–19). The TS is a soft dynamic proprioceptive orthotic (including vest, shorts, knee pads, and specially adapted shoes) that aligns the body by placing pressure on specific areas through a system of interconnected elastic cords (13). The TS was created from a prototype developed for Russian astronauts to counter the effects of long-term weightlessness on the body while in space (13, 16, 20). The elastic cords are systematically adjusted based on the individual needs and functional limitations of the child (16). The TS Method was designed to be used in intensive rehabilitation programs, including vigorous strengthening and stretching exercises, and training of specific motor activities (13). However, despite the popularity of short intensive TS therapy interventions, a recent systematic review and meta-analysis showed they have small effects on the functional skills of children and adolescents with CP (21). The authors raised concern about the lack of evidence for the beneficial effects of this dynamic orthosis, and suggested that specific immediate effects of TS therapy on postural control and gait should be analyzed. The aim of this study is to determine the immediate effects of wearing TS on joint angle displacements in both lower limbs during gait in children with US-CP. The results from this analysis may provide evidence to support the applicability of TS in physical therapy interventions, more specifically on gait training programs.

## Materials and Methods

### Participants

Seven participants (3 females and 4 males, mean age = 7.17 ± 1.01 yrs; ranging from 5 to 9 years) with a medical diagnosis of Unilateral Spastic Cerebral Palsy, level I and II of the Gross Motor Function Classification System (GMFCS), were evaluated using a quasi-experimental study design with one pre-test condition (baseline) and two post-test conditions (Therasuit without elastics and Therasuit with elastics). Participants 1, 2, and 3 had a medical diagnosis of left hemiparesis, and participants 4, 5, 6, and 7 had a medical diagnosis of right hemiparesis.

Inclusion criteria were: (a) unilateral spastic cerebral palsy; (b) ability to walk independently (level I or II according to GMFCS); (c) age range from 5 to 10 years old; (d) cognitive level and emotional state facilitating understanding and cooperation of the participant; (e) no prior experience with this type of dynamic orthosis (TS) before this study. Exclusion Criteria were: (a) another medical diagnosis, types and sub-types of spastic CP; (b) congenital heart disease and cardiorespiratory problems; (c) presence of structural deformities at the lower limbs and trunk, or instability in the ankle joint, which could compromise the child's safety and performance of the motor task; (d) epilepsy; (e) treatment with botulinum toxin in the calf muscles within the previous 6 months; (f) surgical intervention (e.g. tendon lengthening in lower limb) within the previous 12 months; (g) muscle tone scored ≥2, according to Modified Ashworth Scale; (h) severe affective or psychiatric impairments; (i) serious vision or hearing problems. The previous clinical history of the participants is presented on Table 1.

**Table 1:** Previous clinical history.

**Participant**	**Gestational age (weeks)**	**Gestational age classification**	**Birth weight (g)**	**Birth weight classification**	**Type of delivery**	**Neonatal intensive care**	**Assisted ventilation > 24 h**	**Age onset of independent gait (months)**
1	41	LT	3,075	NBW	V	No	No	24
2	34	MLPT	1,845	LBW	V	Yes	Yes	24
3	42	LT	2,500	NBW	SC	No	No	24
4	25	EXPT	833	ELBW	V	Yes	Yes	24
5	30	VPT	1,420	VLBW	EC	Yes	Yes	24
6	25	EXPT	800	ELBW	V	Yes	Yes	30
7	37	ET	2,660	NBW	SC	No	No	24

*LT, Late Term; MLPT, moderate to late preterm; EXPT, extremely preterm; VPT, very preterm; ET, Early Term; NBW, Normal birth weight; LBW, Low birth weight; ELBW, extremely low birth weight; VLBW, very low birth weight; V, Vaginal; SC, Scheduled Cesarean; EC, Emergency Cesarean*.

The Ethical Committee of the Rehabilitation Medicine Center in Alcoitão (CMRA), Portugal (PT) ensured and approved the conformity procedures regarding scientific research involving human beings. Parental consents and children assents were obtained. A convenience sample was used. Children that were attending the CMRA and who met the inclusion criteria were invited to participate.

### Procedure

#### Data Collection

The protocol included two phases of data collection: (1) parental interview and clinical examination; (2) gait analyses in three different conditions: baseline (before TS), TS without elastics (TSWE) and complete TS, with elastics (TS). The option of testing two TS conditions was necessary because for gait analysis some markers that were placed on the skin in baseline condition required placement on the orthosis in the TS condition. In order to avoid a possible systematic bias due to placement of the markers on the orthosis the TSWE was included. However, the condition TSWE could not be considered a baseline condition because, even without elastics the TS may influence the gait patterns of the children since its fabric is not as soft and flexible as other orthosis (e.g., the TTs lycra).

##### Parental interview and clinical examination

The parental interview aimed to collect data regarding pre- and peri-natal history and developmental milestones (Table 1). The diagnosis of US-CP was confirmed by a pediatric neurologist and magnetic resonance exams. Data about orthopedic surgeries and pharmacological treatments of spasticity were also collected. All children had been submitted to at least one application of botulinum toxin (BTX), type A, in the paretic lower limb, with only one child (participant 1) undergoing a muscle stretching in the paretic lower limb (15 months prior to the study).

To understand the health conditions associated with CP and the impact on the functional activity, the clinical examination was performed by two-experienced pediatric physical therapists a week before gait analysis.

A bilateral goniometric assessment of passive range of movement (ROM) was performed for both lower limbs by the same trained physical therapist (22, 23) (see Supplementary Table 1), and the spasticity score of the paretic LL (PLL) was determined according to the Modified Ashworth's Scale (MAS) (24), (see Supplementary Table 2). All the participants showed no structural deformities or instability in the ankle joint of the PLL (dorsiflexion/plantarflexion range from 15° to −15°), which could compromise the child's safety and performance of the motor task and muscle tone scored <2. The length of the lower limbs was clinically assessed (distance from anterior superior iliac spine to medial malleolus).

The functional profiles (Table 2) were based on validated tools to measure and classify the functional severity, according to the recommendation of *Surveillance of Cerebral Palsy in Europe* (SCPE) (25, 26) and the International Classification of Functioning, Disability and Health–Children & Youth (CIF-CY) conceptual model (27).

**Table 2:** Functional Profile and associated disorders, according to SCPE (25, 26) and CIF-CY (27).

**Participant**	**GMFCS**	**BFMF**	**MACS**	**VSS**	**CFCS**	**EDACS**	**Epilepsy**	**Cognitive**	**Education**
1	II	III	III	I	I	I	I	I	II
2	II	III	III	II	II	I	I	II	II
3	I	III	III	III	III	III	I	II	II
4	I	II	II	I	I	I	I	I	II
5	I	II	II	II	II	I	I	II	II
6	II	III	III	II	II	II	I	II	II
7	I	II	II	I	I	I	I	I	I

*CMFCS, Global Motor Function Classification System (28); MACS, Manual Ability Classification System(29); BFMF, Bilateral Fine Motor Function (30); VSS, Viking Speech Scale (31); CFCS, Communication Function Classification System (32); EDACS, Eating and Drinking Classification System (33); Associated disorders and education (school participation) evaluated according to Cans (25) and Andrada et al. (26)*.

The gross motor function, balance assessments and functional mobility (Table 3), were performed using the Gross Motor Function Measure (GMFM-66 items) (34). Functional Mobility Score (FMS) (35, 36), and the Pediatric Balance Scale (PBS) (37) Additionally, a video gait analysis was done, to assess the gait patterns of each participant in the sagittal plane, based in the classification proposed by Winters, Gage and Hicks (see Supplementary Table 3) (38). ROM and MAS scores and the observational gait analysis were used to support the decision-making process regarding the placement of the elastic cords in each of the seven children (see Supplementary Table 4).

**Table 3:** Gross motor function, balance and functional mobility.

**Participant**	**GMFM (%)**	**PBS**	**FMS**		
			**5 m**	**50 m**	**500 m**
1	94	43/56	6	6	5
2	94	43/56	6	6	5
3	96	44/56	6	6	5
4	97	45/56	6	6	5
5	96	45/56	6	6	5
6	94	43/56	6	5	5
7	98	52/56	6	6	6

*GMFM, Gross Motor Function Measure (66 items) (34); PBS, Pediatric Balance Scale (37); FMS, Functional Mobility Score (35, 36)*.

##### Gait analysis protocol

A week after the clinical examination, 3D gait analysis following a standardized protocol was conducted in three different conditions: (A) baseline (BL); (B) while wearing TS without elastics (TSWE); and (C) while wearing the complete TS (TS). The assessments were performed in 1 day by two experienced pediatric physical therapists, one of them with TS certification. The order of the conditions was the same for all children (i.e., baseline, TSWE, TS), increasing gradually the complexity of the orthosis, in order to shorten the total data collection time and to avoid possible stress or fatigue related with dressing and undressing the TS. Data from clinical examination (ROM and MAS) and observational gait analysis were used to guide the placing of the elastic cords of the TS according to each participant's specific needs. The participant's assent was obtained after an initial explanation prior to the data collection procedures.

Two video-digital cameras (Basler piA1000-48gc GigE) and six infrared cameras (VICON T10) sampled at 100 Hz in conjunction with four force-plates (AMTI OR6-7-2000) and four analog AMTI amplifiers were used to measure 3D motion of the lower limbs (LLs).

Anthropometric measures (height, body weight, tibial length, distance between the femoral condyles or diameter of the knee, distance between the *malleoli* or diameter of the ankle, distance between the anterior iliac spines and thickness of the pelvis) were collected in the two conditions. Height and weight were collected with shoes on in both conditions.

After collecting the anthropometric measures, in the BL condition sixteen retro-reflective markers were fixed with double-sided adhesive tape directly on the skin in anatomical landmarks of the pelvis and lower extremities identified by palpation. The placement of the markers in these anatomical landmarks allows the tracking of each functional segment's movement trajectory. In the TSWE and TS conditions, the markers were placed on the dynamic orthosis in positions that best reproduced the original anatomical references used in the BL condition (see Supplementary Figure 1). In some cases (e.g., pelvis) each marker was moved laterally by an equal distance along the ASIS-ASIS axis. The true inter-ASIS distance was then manually measured and entered in Vicon, following the Plug-in Gait model recommendations. In the thigh and leg segments, rigid clusters were also used according to the Lowerbody Vincon Model (Plug-in Gait) (39, 40). To avoid marker displacement between the BL and both TS conditions, and to reduce the influence of the footwear on the gait pattern, all participants used the same model of commercially available sneakers during the test protocol (41). The markers were placed in anatomical landmarks equivalent to those reported in the literature (42). For the hallux and heel, the markers were placed on the shoe in positions that best reproduced the reported barefoot anatomical references (42).

After placing the markers, a reference position was captured to determine the overall position and orientation of the markers within the body segments (43). To this end, the participants were instructed to remain in an orthostatic position with their feet aligned in the center of the force platform. The reference position was determined after collecting the marker data for 5 s in each condition. Next, the participants were instructed to walk up and down at a comfortable walking speed until they were asked to stop, in order to perform a total of 10 trials (about 20 m). Kinematic variables were recorded until at least five successful trials (range: 5–8) for each lower limb in each condition. Rest breaks of 20 min were allowed between conditions.

Different outcome variables regarding the kinematic measures of lower limbs in the sagittal plane were analyzed in this study (Table 4).

**Table 4:** Kinematic measures of lower limbs in sagittal plane.

**Joint**	**Description**
Hip joint (flexion/extension)	- Hip initial contact: value of hip angle at initial contact; - Hip angle at mid-stance: minimum of hip flexion in mid-stance sub-phase; - Hip angle at mid- swing: peak of hip flexion at mid swing sub-phase;
Knee joint (flexion/extension)	- Knee angle at initial contact: value of knee angle at initial contact; - Knee angle at mid-stance: minimum of knee flexion at mid-stance sub-phase; - Knee angle at mid-swing: peak of knee flexion at mid- swing sub-phase;
Ankle joint (dorsi/plantar flexion)	- Ankle angle at initial contact: value of the ankle joint angle at the initial contact. - Ankle angle at mid-stance: ankle joint angle at mid-stance sub-phase; - Ankle angle at swing phase: value of the ankle joint angle at mid-swing phase.

#### Data Analysis

Acquisition and processing of the biomechanical data were performed with the Vicon® system by using the software Nexus version 1.8.5. We analyzed the kinematic data with the Plug-in-Gait model (44), which is widely used in clinical gait analysis (45). The Polygon version 3.5.2. software was used for the analysis. All data used in this analysis was previously archived in folders and exported to Excel files. Each file stored task and evaluation data corresponding to one participant.

A descriptive exploratory analysis was carried out to identify aspects or behavior patterns characterizing the variables under study. The Wilcoxon test for paired samples, with Bonferroni correction, was performed to compare the mean ranks of each variable in the BL, TSWE and TS conditions. SPSS 24 was used for the statistical analyses with a *p* < 0.05 as the level of statistical significance. However, the *p* value cannot provide full information about the practical significance of the results or about whether or not the result is replicable. In studies with small samples the *p*-value could not reflect if the underlying difference is real. We therefore used the Z-scores to calculate correlation coefficients (46) and to quantify the effect sizes of the TS therapy. Values were considered small if *r* ≥ 0.10; moderate, if *r* ≥ 30 and large if *r* ≥ 0.50 (47–49).

## Results

### Hip Joint

**Figure 1:**
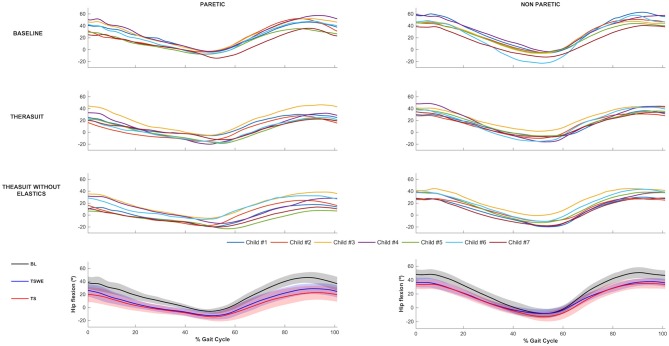
Hip joint flexion-extension. Top 3 rows represent the angular displacements in the seven children during a gait cycle, in the three conditions, for the PLL and NPLL. Bottom row represents the group average and SD values (shaded areas) in the three conditions, for the PLL and NPLL. Negative values represent hip extension.

The hip joint sagittal plane angular displacements in the seven children during a gait cycle, as well as the group average and SD values in the three conditions, for the PLL and NPLL, are presented in Figure 1. All participants showed a tendency to have lower hip flexion pattern in the TS conditions, in both lower limbs during the gait cycle.

Table 5 presents the comparison for the hip joint kinematic variables between conditions during the different stages of gait cycle.

**Table 5:** Comparison for the hip joint kinematic variables between conditions, during the different stages of gait cycle, in both lower limbs (Wilcoxon test with Bonferroni correction and effect size).

	**Kinematic variables/Gait Phases/Condition**	**Mean**	**SD**	**Wilcoxon/Bonferroni Correction**	**Effect size**
		**(**°**)**	**(**°**)**	(^*^p < 0.017)	**(*r* = Z/ $\sqrt{n}$)**
Paretic lower limb	1-Hip initial contact BL	37.99	9.45	1 vs. 2; *Z* = −2.366, *p* = 0.008*	1 vs. 2; *r* = −0.63
	2-Hip initial contact TSWE	26.36	9.51	1 vs. 3; *Z* = −2.366, *p* = 0.008*	1 vs. 3; *r* = −0.63
	3-Hip initial contact TS	19.84	11.78	2 vs. 3; *Z* = −1.859, *p* = 0.039	2 vs. 3; *r* = −0.50
	1-Hip extension angle stance phase BL	−6.31	4.07	1 vs. 2; *Z* = −1.859, *p* = 0.039	1 vs. 2; *r* = −0.50
	2-Hip extension angle stance phase TSWE	−13.40	6.09	1 vs. 3; *Z* = −2.201, *p* = 0.016*	1 vs. 3; *r* = −0.59
	3-Hip extension angle stance phase TS	−15.30	6.65	2 vs. 3; *Z* = −0.676, *p* = 0.289	2 vs. 3; *r* = −0.18
	1-Hip flexion angle swing phase BL	46.57	8.34	1 vs. 2; *Z* = −2.366, *p* = 0.008*	1 vs. 2; *r* = −0.63
	2-Hip flexion angle swing phase TSWE	29.49	8.18	1 vs. 3; *Z* = −2.366, *p* = 0.008*	1 vs. 3; *r* = −0.63
	3-Hip flexion angle swing phase TS	23.20	10.91	2 vs. 3; *Z* = −1.778, *p* = 0.047	2 vs. 3; *r* = −0.48
	1-Hip range of movement BL	52.84	5.50	1 vs. 2; *Z* = 2.366, *p* = 0.008*	1 vs. 2; *r* = −0.63
	2-Hip range of movement TSWE	42.93	7.04	1 vs. 3; *Z* = −2.366, *p* = 0.008*	1 vs. 3; *r* = −0.63
	3-Hip range of movement TS	39.07	5.50	2 vs. 3; *Z* = −1.778, *p* = 0.047	2 vs. 3; *r* = −0.48
Non-paretic lower limb	1-Hip initial contact BL	48.34	7.23	1 vs. 2; *Z* = −2.366, *p* = 0.008*	1 vs. 2; *r* = −0.63
	2-Hip initial contact TSWE	36.77	6.73	1 vs. 3; *Z* = −2.366, *p* = 0.008*	1 vs. 3; *r* = −0.63
	3-Hip initial contact TS	34.06	6.56	2 vs. 3; *Z* = −1.183, *p* = 0.148	2 vs. 3; *r* = −0.32
	1-Hip extension angle stance phase BL	−8.66	6.88	1 vs. 2; *Z* = 0.000, *p* = 0.531	1 vs. 2; *r* = 0.00
	2-Hip extension angle stance phase TSWE	−8.76	6.05	1 vs. 3; Z = −1.270, p = 0.117	1 vs. 3; *r* = −0.34
	3-Hip extension angle stance phase TS	−13.09	6.70	2 vs. 3; *Z* = −1.690, *p* = 0.055	2 vs. 3; *r* = −0.45
	1-Hip flexion angle swing phase BL	51.46	8.19	1 vs. 2; *Z* = −2.366, *p* = 0.008*	1 vs. 2; *r* = −0.63
	2-Hip flexion angle swing phase TSWE	37.96	5.05	1 vs. 3; *Z* = −2.366, *p* = 0.008*	1 vs. 3; *r* = −0.63
	3-Hip flexion angle swing phase TS	35.76	6.84	2 vs. 3; *Z* = −1.270, *p* = 0.117	2 vs. 3; *r* = −0.34
	1-Hip range of movement BL	60.57	10.72	1 vs. 2; *Z* = −2.197, *p* = 0.016 *	1 vs. 2; *r* = −0.59
	2-Hip range of movement TSWE	47.90	9.27	1 vs. 3; *Z* = −2.197, *p* = 0.016*	1 vs. 3; *r* = −0.59
	3-Hip range of movement TS	48.96	6.07	2 vs. 3; *Z* = −0.676, *p* = 0.289	2 vs. 3; *r* = −0.18

*BL, Baseline; TSWE, Therasuit without elastics; TS, Therasuit*.

Statistically significant differences between conditions were observed on the PLL at the different gait stages, namely: (i) hip angle at initial contact between BL and both TS conditions (*p* = 0.008, *r* = 0.63), (ii) hip extension angle at mid-stance between BL and TS (*p* = 0.016, *r* = 0.59); iii) hip flexion angle at mid-swing between BL and both TS conditions (*p* = 0.008, *r* = 0.63).

For the NPLL, differences were found at the following stages: (i) hip angle at initial contact between BL and both TS conditions (*p* = 0.008, *r* = 0.63) and; (ii) hip flexion angle at mid-swing between BL and both TS conditions (*p* = 0.008, *r* = 0.63).

Regarding ROM, significant differences were found between BL and both TS conditions at the hip of the PLL (*p* = 0.008, *r* = 0.63) and of the NPLL (*p* = 0.016, *r* = 0.59).

Despite the statistically non-significant differences, there were also large effect sizes in the PLL at: (i) initial contact between the two TS conditions (*p* = 0.039, *r* = 0.50); (ii) stance phase between BL and TSWE (*p* = 0.039, *r* = 0.50).

### Knee Joint

**Figure 2:**
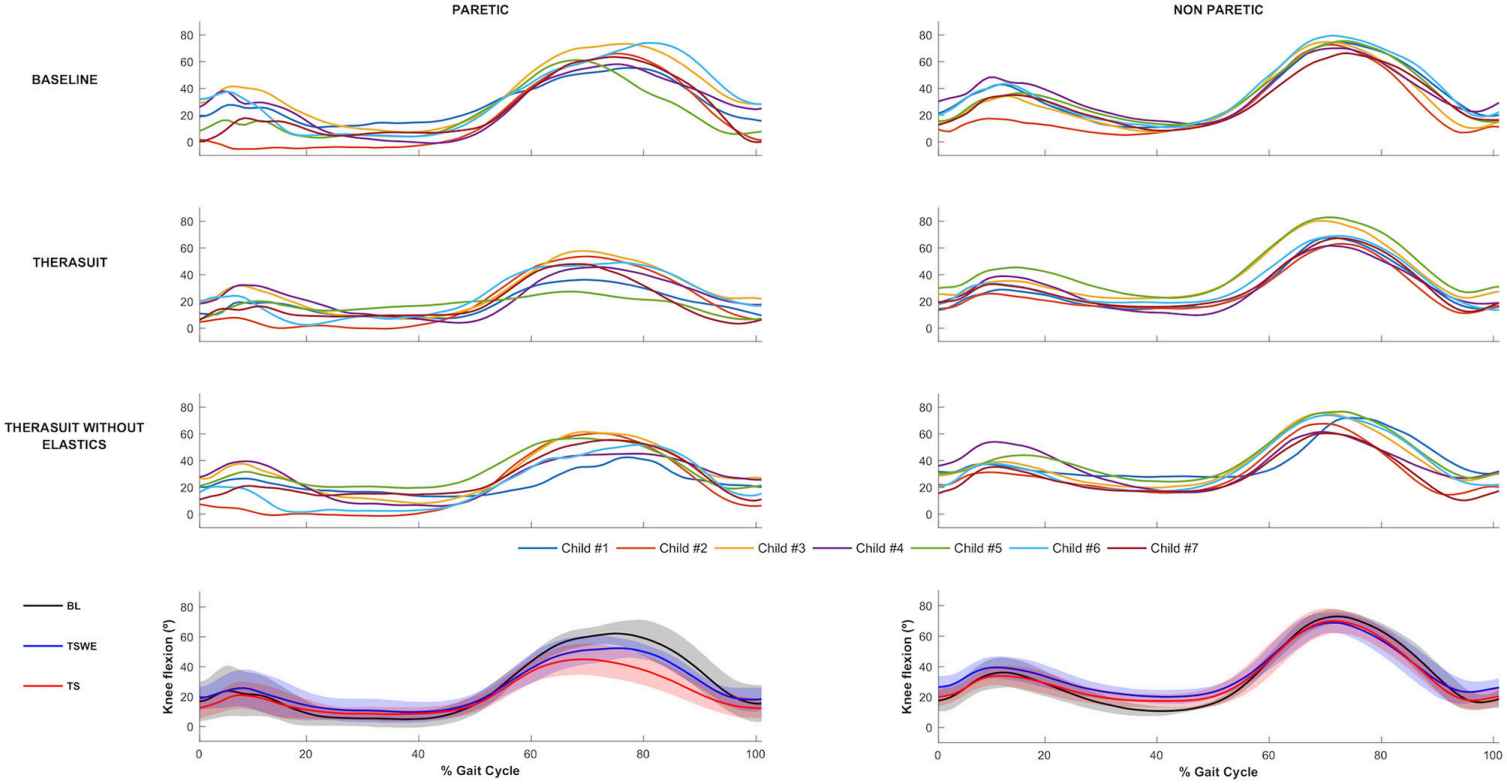
Knee joint flexion-extension Top 3 rows represent the angular displacements in the seven children during a gait cycle, in the three conditions, for the PLL and NPLL. Bottom row represents the group average and SD values (shaded areas) in the three conditions, for the PLL and NPLL. Negative values represent knee extension.

The knee joint sagittal plane angular displacements in all participants during a gait cycle and also the group average and SD values in the three conditions, for the PLL and NPLL, are presented in Figure 2. In contrast to the hip joint, the seven participants showed high heterogeneity or inter-individual variability in knee joint motor patterns in the BL condition, which is possibly related to the lower limb discrepancy between the PLL and the NPLL exhibited by the children.

Table 6 presents the comparison for the knee joint kinematic variables between conditions during the different stages of gait cycle.

**Table 6:** Comparison for the knee joint kinematic variables between conditions, during the different stages of gait cycle, in both lower limbs (Wilcoxon test with Bonferroni correction and effect size).

	**Kinematic variables/Gait phases/Condition**	**Mean**	**SD**	**Wilcoxon/Bonferroni correction**	**Effect size**
		**(**°**)**	**(**°**)**	(*p < 0.017)	**(*r* = Z/ $\sqrt{n}$)**
Paretic lower limb	1-Knee initial contact BL	16.67	13.26	1 vs. 2; *Z* = −0.676, *p* = 0.289	1 vs. 2; *r* = −0.18
	2-Knee initial contact TSWE	18.77	7.63	1 vs. 3; *Z* = −1.521, *p* = 0.078	1 vs. 3; *r* = −0.41
	3-Knee initial contact TS	12.33	6.86	2 vs. 3; *Z* = −2.197, *p* = 0.016*	2 vs. 3; *r* = −0.59
	1-Knee extension angle stance phase BL	2.80	5.37	1 vs. 2; *Z* = 1.859, *p* = 0.039	1 vs. 2; *r* = −0.50
	2-Knee extension angle stance phase TSWE	8.36	7.05	1 vs. 3; *Z* = −1.185, *p* = 0.141	1 vs. 3; *r* = −0.32
	3-Knee extension angle stance phase TS	4.67	2.87	2 vs. 3; *Z* = −1.859, *p* = 0.039	2 vs. 3; *r* = −0.50
	1-Knee flexion angle swing phase BL	64.57	7.16	1 vs. 2; *Z* = −2.366, *p* = 0.008*	1 vs. 2; *r* = −0.63
	2-Knee flexion angle swing phase TSWE	53.46	7.29	1 vs. 3; *Z* = −2.366, *p* = 0.008*	1 vs. 3; *r* = −0.63
	3-Knee flexion angle swing phase TS	45.31	10.42	2 vs. 3; *Z* = −2.197, *p* = 0.016*	2 vs. 3; *r* = −0.59
	1-Knee range of movement BL	61.83	9.15	1 vs. 2; *Z* = 2.366, *p* = 0.008*	1 vs. 2; *r* = −0.63
	2-Knee range of movement TSWE	45.29	10.87	1 vs. 3; *Z* = −2.371, *p* = 0.008*	1 vs. 3; *r* = −0.63
	3-Knee range of movement TS	41.09	12.03	2 vs. 3; *Z* = −1.778, *p* = 0.047	2 vs. 3; *r* = −0.48
Non-paretic lower limb	1- Knee extension angle stance phase BL	9.86	2.94	1 vs. 2; *Z* = −2.366, *p* = 0.008*	1 vs. 2; *r* = −0.63
	2-Knee extension angle stance phase TSWE	19.66	4.72	1 vs. 3; *Z* = −2.028, *p* = 0.023	1 vs. 3; *r* = −0.56
	3-Knee extension angle stance phase TS	16.21	4.86	2 vs. 3; *Z* = −1.521, *p* = 0.078	2 vs. 3; *r* = −0.41
	1-Knee initial contact BL	17.63	7.01	1 vs. 2; *Z* = −2.371, *p* = 0.008*	1 vs. 2; *r* = −0.63
	2-Knee initial contact TSWE	26.49	7.17	1 vs. 3; *Z* = −0.593, *p* = 0.305	1 vs. 3; *r* = −0.16
	3-Knee initial contact TS	19.84	5.87	2 vs. 3; *Z* = −1.778, *p* = 0.047	2 vs. 3; *r* = −0.48
	1-Knee flexion angle swing phase BL	73.36	4.18	1 vs. 2; *Z* = −1.859, *p* = 0.039	1 vs. 2; *r* = −0.50
	2-Knee flexion angle swing phase TSWE	69.76	6.66	1 vs. 3; *Z* = −1.183 *p* = 0.148	1 vs. 3; *r* = −0.32
	3-Knee flexion angle swing phase TS	70.24	8.22	2 vs. 3; *Z* = −0.593. *p* = 0.313	2 vs. 3; *r* = −0.16
	1-Knee range of movement BL	63.50	4.68	1 vs. 2; *Z* = −2.366, *p* = 0.008*	1 vs. 2; *r* = −0.63
	2-Knee range of movement TSWE	51.09	4.86	1 vs. 3; *Z* = −2.366, *p* = 0.008*	1 vs. 3; *r* = −0.63
	3-Knee range of movement TS	55.26	3.07	2 vs. 3; *Z* = −1.863, *p* = 0.039	2 vs. 3; *r* = −0.50

*BL, Baseline; TSWE, Therasuit without elastics; TS, Therasuit*.

Statistically significant differences for the PLL were found at the following stages of the gait cycle: (i) knee angle at initial contact between both TS conditions (*p* = 0.016, *r* = 0.59); (ii) knee flexion angle at mid-swing between BL and both TS conditions (*p* = 0.008, *r* = 0.63) and between both TS conditions (*p* = 0.016, *r* = 0.59).

For the NPLL differences were found at the following stages: (i) knee angle at initial contact between BL and TSWE (*p* = 0.008, *r* = 0.63), (ii) knee angle at mid-stance between BL and TSWE (*p* = 0.008, *r* = 0.63).

Regarding ROM, there were differences between BL and both TS conditions in the PLL (*p* = 0.008, *r* = 0.63) and in the NPLL (*p* = 0.008, *r* = 0.63).

Despite the lack of statistically significant differences, large effect sizes were observed at knee extension at mid-stance in both lower limbs. In the PLL large effects were observed between BL and TSWE (*p* = 0.039, *r* = 0.50), and between both TS conditions (*p* = 0.039, *r* = 0.50). In the NPLL, still at stance phase, large effect sizes were found between BL and TS (*p* = 0.023, *r* = 0.56). In swing phase, large effect sizes were noted between the BL and TSWE (*p* = 0.039, *r* = 0.50). Large effect sizes were also noted between the two TS conditions in the ROM of the NPLL (*p* = 0.039, *r* = 0.50).

### Ankle Joint

**Figure 3:**
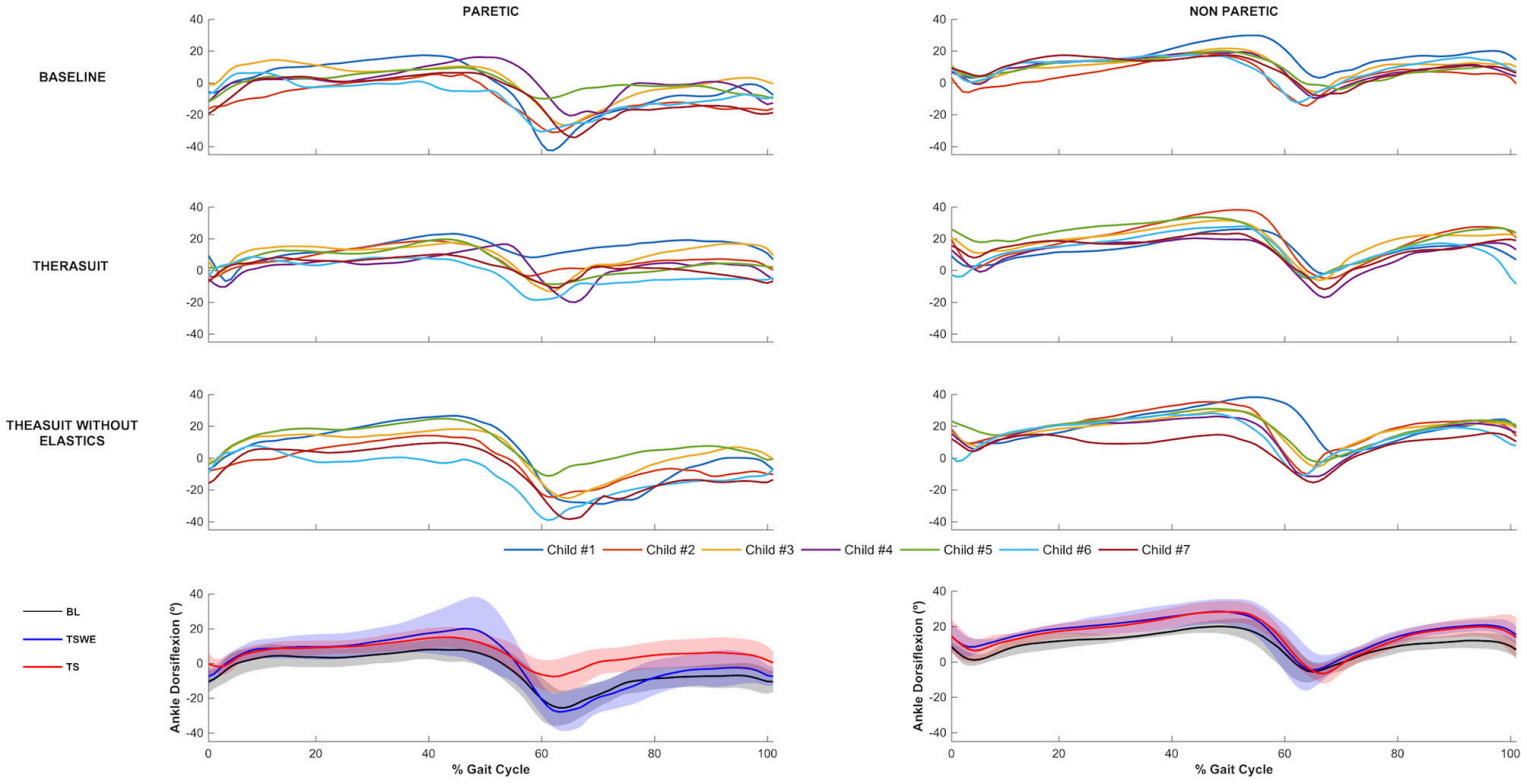
Ankle joint flexion-extension. Top 3 rows represent the angular displacements in the seven children during a gait cycle, in the three conditions, for the PLL and NPLL. Bottom row represents the group average and SD values (shaded areas) in the three conditions, for the PLL and NPLL. Negative values represent plantarflexion.

The ankle joint sagittal plane angular displacements in all participants during a gait cycle, plus the group average and SD values in the three conditions, for the PLL and NPLL, are presented in Figure 3. Although all participants had similar kinematic patterns in the ankle joints, there were differences between the PLL and NPLL at different phases of the gait cycle.

Table 7 presents the comparison for the ankle joint kinematic variables between conditions during the different stages of gait cycle.

**Table 7:** Comparison for the ankle joint kinematic variables between conditions, during the different stages of gait cycle, in both lower limbs (Wilcoxon test with Bonferroni correction and effect size).

	**Kinematic variables/Gait phases/Condition**	**Mean**	**SD**	**Wilcoxon/Bonferroni correction**	**Effect size**
		**(**°**)**	**(**°**)**	(*p < 0.017)	**(*r* = Z/ $\sqrt{n}$)**
Paretic lower limb	1-Ankle initial contact BL	−10.49	6.31	1 vs. 2; Z = −1.521, *p* = 0.078	1 vs. 2; *r* = −0.41
	2-Ankle initial contact TSWE	−7.47	4.72	1 vs. 3; *Z* = −2.366, *p* = 0.008*	1 vs. 3; *r* = −0.63
	3-Ankle initial contact TS	−0.29	6.16	2 vs. 3; *Z* = −2.366, *p* = 0.008*	2 vs. 3; *r* = −0.63
	1-Ankle dorsiflexion stance phase BL	4.53	4.87	1 vs. 2; *Z* = −2.197, *p* = 0.016*	1 vs. 2; *r* = −0.59
	2-Ankle dorsiflexion stance phase TSWE	10.01	7.97	1 vs. 3; *Z* = −2.366, *p* = 0.008*	1 vs. 3; *r* = −0.63
	3-Ankle dorsiflexion stance phase TS	11.66	3.36	2 vs. 3; *Z* = −0.676, *p* = 0.289	2 vs. 3; *r* = −0.18
	1-Ankle angle swing phase BL	−9.44	7.25	1 vs. 2; *Z* = −0.507, *p* = 0.344	1 vs. 2; *r* = −0.14
	2-Ankle angle swing phase TSWE	−11.07	10.19	1 vs. 3; *Z* = −2.197, *p* = 0.016*	1 vs. 3; *r* = −0.59
	3-Ankle angle swing phase TS	3.86	7.89	2 vs. 3; *Z* = −2.197, *p* = 0.016*	2 vs. 3; *r* = −0.59
	1-Ankle range of movement BL	39.11	11.26	1 vs. 2; *Z* = −1.859, *p* = 0.039	1 vs. 2; *r* = −0.50
	2-Ankle range of movement TSWE	52.39	21.27	1 vs. 3; *Z* = −2.028, *p* = 0.023	1 vs. 3; *r* = −0.54
	3-Ankle range of movement TS	28.14	5.14	2 vs. 3; *Z* = −2.366, *p* = 0.008*	2 vs. 3; *r* = −0.63
Non-Paretic lower limb	1-Ankle initial contact BL	8.56	2.73	1 vs. 2; *Z* = −1.521, *p* = 0.078	1 vs. 2; *r* = −0.41
	2-Ankle initial contact TSWE	14.49	7.02	1 vs. 3; *Z* = −1.352, *p* = 0.109	1 vs. 3; *r* = −0.36
	3-Ankle initial contact TS	14.43	9.37	2 vs. 3; *Z* = −0.169, *p* = 0.469	2 vs. 3; *r* = −0.05
	1-Ankle dorsiflexion stance phase BL	13.70	1.89	1 vs. 2; *Z* = −2.201, *p* = 0.016*	1 vs. 2; *r* = −0.59
	2-Ankle dorsiflexion stance phase TSWE	22.19	6.10	1 vs. 3; *Z* = −2.201, *p* = 0.016*	1 vs. 3; *r* = −0.59
	3-Ankle dorsiflexion stance phase TS	20.79	5.05	2 vs. 3; Z = −0.676, p = 0.289	2 vs. 3; *r* = −0.18
	1-Ankle angle swing phase BL	9.90	3.31	1 vs. 2; *Z* = −2.197, *p* = 0.016*	1 vs. 2; *r* = −0.59
	2-Ankle angle swing phase TSWE	16.10	3.35	1 vs. 3; *Z* = −2.197, *p* = 0.016*	1 vs. 3; *r* = −0.59
	3-Ankle angle swing phase TS	15.64	3.55	2 vs. 3; *Z* = −0.423, *p* = 0.367	2 vs. 3; *r* = −0.11
	1-Ankle range of movement BL	27.87	2.96	1 vs. 2; *Z* = −2.366, *p* = 0.008*	1 vs. 2; *r* = −0.63
	2-Ankle range of movement TSWE	37.07	4.60	1 vs. 3; *Z* = −2.366, *p* = 0.008*	1 vs. 3; *r* = −0.63
	3-Ankle range of movement TS	36.64	4.51	2 vs. 3; *Z* = 0.000, *p* = 0.531	2 vs. 3; *r* = 0.00

*BL, Baseline; TSWE, Therasuit without elastics; TS, Therasuit*.

The results show significant differences on the PLL at different stages of gait cycle, namely: (i) ankle angle at initial contact between BL and TS (*p* = 0.008, *r* = 0.63) and between both TS conditions (*p* = 0.008, *r* = 0.63); (ii) dorsiflexion angle at stance phase between BL and both TS conditions (TSWE: *p* = 0.016, *r* = 0.59; TS: *p* = 0.008, *r* = 0.63); (iii) dorsiflexion angle at swing phase between BL and TS (*p* = 0.016, *r* = 0.59) and between both TS conditions (*p* = 0.016, *r* = 0.59).

Regarding the NPLL, there were significant differences only at the following stages of gait cycle: (i) dorsiflexion angle at stance phase between BL and both TS conditions (*p* = 0.016, *r* = 0.59); (ii) dorsiflexion angle at swing phase between BL and both TS conditions (*p* = 0.016, *r* = 0.59).

Concerning ankle ROM in the PLL, there were differences between both TS conditions (*p* = 0.008, *r* = 0.63). In the NPLL, there were differences in ankle ROM between BL and both TS conditions (*p* = 0.008, *r* = 0.63).

Despite the lack of statistically significant differences, there were large effect sizes at ROM of the PLL between BL and both TS conditions (TSWE: *p* = 0.039, *r* = 0.50; TS: *p* = 0.023, *r* = 0.54).

## Discussion

This study assessed the immediate effects of wearing Therasuit on the angular displacements of the lower limbs during gait in children with unilateral spastic cerebral palsy (US-CP). Overall the TS showed some significant effects in the three analyzed joints. However, the positive results in the ankle are more reliable, since in this joint, markers were not moved between conditions, eliminating any possible systematic bias due to the placement of the markers between conditions. The discussion of the effects noted in the hip, knee and ankle joints is presented below.

The results showed that wearing a Therasuit (with or without elastics) has statistically significant effects and large effect sizes on most of the angular hip variables in both lower limbs, correcting the exaggerated flexion pattern during the whole gait cycle in all participants. Even though there were no statistically significant differences between both Therasuit conditions in this joint, the results were most notable on the TS condition than TSWE in the PLL, resulting in lower hip flexion pattern during whole gait cycle.

Children with US-CP often have an exaggerated hip flexion pattern during gait to compensate for motor control impairments in the PLL and for leg length discrepancy (i.e., longer NPLL) (50). Our results suggest that the Therasuit helps to reduce this greater hip flexion pattern during the gait cycle, and this is consistent with previous data showing an increase in hip extension at stance phase in children with bilateral spastic (diplegia) CP, wearing the Theratogs dynamic orthosis (51). In our study, the Therasuit promoted a greater hip extension pattern at initial contact in both lower limbs and at mid-stance sub-phase in the PLL and lower hip flexion pattern in both lower limbs, at mid-swing, resulting in a tendency to approach normative angular values for this joint (52). This effect is likely due to the decrease of the musculoskeletal constraints on the hip gait patterns in the PLL. According to Hussein et al. (53), the decrease in the typical spasticity flexion pattern allows agonist and antagonist muscles to work in better synchrony, leading to a smother movement.

The results in the knee joint were less clear than in the hip, probably also due to a greater heterogeneity between children. In the PLL, most relevant differences were found in swing phase, since the exaggerated flexion pattern exhibited in BL decreased toward normative values in the TS conditions, with significant differences and large effect sizes between the three conditions. Regarding the NPLL, there were significant differences on the knee angle at initial contact and stance phase, between BL and TSWE, with large effect sizes. Other large effect sizes (without statistically significant differences) were found on the knee extension angle at stance-phase between both TS conditions and on the knee flexion angle at swing phase between BL and TSWE conditions. One possible explanation for these results, may be related with the high inter-individual variability exhibited by the seven participants in the baseline condition, regarding the knee joint motor patterns in the PLL during the stance phase. This variability was especially visible at mid-stance, ranging from full extension angles similar to children with typical development (Child 4, Child 5, Child 6, and Child 7), to hyperextension (Child 2) and flexion patterns (Child 1 and Child 3), usually showed in gait patterns in children with US-CP. Considering the within participant changes, the TS seemed to help to correct the hyperextension pattern of Child 2 and had slightly positive effects on children with flexion pattern (Child 1 and Child 3).

Furthermore, all the participants have lower limbs length discrepancy and four of them (Child 1, Child 2, Child 6, and Child 7) have the PLL significantly shorter than the NPLL (54). This clinically significant (≥1.5 cm) lower limb length discrepancy (55) may have limited the effects of the Therasuit, more specifically, on the hip and knee joints angles in the NPLL at stance phase.

The fact that there were almost no significant differences between TSWE and TS conditions on the hip and knee joints might be related with the biomechanical constraints caused by the TS fabric.

Regarding the ankle joint, the results showed significant differences and large effect sizes on all kinematic variables in the PLL between BL and TS with elastics, during whole gait cycle. These differences with BL only occurred in stance phase for the TSWE condition. Comparing both TS conditions, the significant differences and large effect sizes were also noted, at initial contact and swing phases. Conversely, in the NPLL, there were significant differences and large effect sizes on the dorsiflexion angles, at stance and swing phases, between BL and both TS conditions.

The ankle joint showed an asymmetrical pattern. While in the baseline condition the ankle joint on the PLL is plantarflexed at initial contact and swing phases, the NPLL is characterized by a greater ankle dorsiflexion pattern, in these phases of the gait cycle. According to our findings, the TS with elastics successfully controlled the excessive ankle plantar flexion (equinus foot) in the PLL at swing phase, correctly pre-positioning the foot for an initial heel contact. This correction was observed in all the participants and led the ankle angles values at initial contact toward the normative values (52), with the exception of participant 1 (the only participant submitted to orthopedic surgery), who exhibited an ankle angle higher than the normative values. In turn, in the NPLL at initial contact in the BL condition, most participants showed a greater dorsiflexion than typically developing children (52), which was maintained in TS conditions. According to Allen et al. (55) and Cimolin et al. (52), the motor control changes observed in most participants in the NPLL, probably result from compensatory strategies used to overcome the structural and functional limitations of the PLL, thereby allowing greater stability over time.

Interestingly, the effects of the Therasuit were also observed on the distal ankle joint. These findings contradict previous research with other dynamic orthoses (i.e., Theratogs) that cause improvements at a proximal level but not at a distal level (56). These conflicting results may be explained by the differences in the samples (participants with bilateral spastic CP at Rennie's study vs. unilateral spastic CP in the present study) or by differences in the orthoses (Theratogs vs. Therasuit). In contrast to Therathogs, the Therasuit allows the correction of the ankle joint by adjusting the elastic cords attached to the shoes, thus augmenting the possibilities to improve distal control.

In summary, our results suggest that wearing a Therasuit could allow a greater extension pattern in the initial contact and stance phases on the PLL (hip and knee joints), as well as a greater dorsiflexion at the ankle joint during the whole gait cycle. These changes promote better dynamic control in the sagittal plane, caused by the reduction of the biomechanical constrains in that limb. These adaptations consequently lead to more functional gait patterns in our sample (seven 6- to 9-year-old children, with US-CP and with minimal degree of spasticity on the PLL).

### Clinical Implications

The (re)habilitation of gait disorders in children with CP is one of the main objectives of physical therapeutic intervention, because the gait has a critical role in the autonomy and quality of life of a child and family (30). The implementation of therapeutic innovations, including Therasuit, into the physical therapist's clinical practice to facilitate more efficient gait patterns in children with US-CP, should be based on a clear understanding of the biomechanical factors underlying the motor and proprioceptive impairments of this subtype of CP. It is also important to note that the inherent complexity of the Therasuit orthosis requires experienced physical therapists for placing the elastic bands in a way that restrains compensatory patterns while simultaneously promoting new and more efficient gait patterns.

This study has important clinical implications since it emphasizes on one hand the presence of differentiated kinematic patterns between the paretic and non-paretic lower limbs (52) and, on other hand, it reinforces the presence of significant compensatory motor strategies in the NPLL that result from neuromuscular factors, biomechanical constraints and primary growth disturbances (e.g., limb length discrepancy) of the PLL (55).

In order to implement an evidence-based practice, the best research evidence should be available. Thus, from a clinical perspective, the identification and precise quantification of gait patterns in both lower limbs (paretic and non-paretic) of children with unilateral spastic CP is a central issue for development of effective and specific (re)habilitation programs. Despite the limitations presented next, the results of the present study may constitute a first step to guide physical therapists in the selection of appropriate dynamic orthoses to promote more functional gait patterns in this particular CP subtype (unilateral spastic).

### Methodological Considerations and Limitations

To our knowledge, this is the first study to investigate the immediate effects of Therasuit on the gait pattern of children with CP. Most studies have addressed the effects of Therasuit interventions (21) and therefore have various confounding variables (e.g., different activities performed during therapy and different intensities of training), making the interpretation of the results challenging and the specific effects of Therasuit difficult to determine. The clinical type and severity level of children with CP influence the functional prognosis and the aims of (re)habilitation programs. For this reason, a previous systematic review has suggested the use of homogeneous samples for studying the effects of Therasuit in the gait pattern of children with CP (21). In this study, we used a homogeneous sample comprising children with similar medical diagnoses (US-CP). Moreover, two experienced pediatric physical therapists performed a detailed physical examination, to ensure similar functional profiles between the participants. Finally, the same two experienced physical therapists were responsible for placing the elastic bands of the TS, in order to ensure the best correction of the musculoskeletal constraints, according to individual needs of each participant.

Nevertheless, this study has some limitations, namely: (1) a convenience sample was used, although the authors had no previous therapeutic relationship with the children until the beginning of the study; (2) the small sample size does not allow the generalization of the results, which should always be made with caution in these type of population; (3) only the sagittal plane was analyzed, hindering the calculus of an index of overall gait pathology, such as the Gait Deviation Index (57) or Gait Profile Score (58), which could help to better understand the clinical significance of the results; (4) the inherent problems associated to the kinematic analysis using markers, namely soft tissue artifact, particularly when not applied directly on the skin in both TS conditions. The placement of some markers on the TS, requires an estimation of the position of anatomical landmarks, which may have produced some error in the results, even being performed by an experienced professional. Due to this, and since we cannot determine the error magnitudes associated with the placement of the markers on the soft tissue, we believe that results regarding the hip and knee joints should be interpreted with extra caution.

### Recommendations for Future Research

Comparative studies with children with unilateral spastic CP are difficult to perform because cortical compromise may generate different patterns of gait. It therefore remains challenging to develop a universal gait classification system for standardizing participant samples and data comparisons between studies (59). The wide variety of interventions used in children with US-CP likely results from the current knowledge gap in what causes the gait pattern changes in these children. Indeed, many of these interventions are insufficiently supported by scientific evidence. Thus, we suggest that future research with more representative samples (i.e., bigger and larger age range samples) should address the immediate and long-term effects of the Therasuit dynamic orthosis on spatial, temporal and angular variables in the three planes (sagittal, frontal, and transverse), not only in this particular CP subtype, but also in other types of CP, such as the dyskinetic type, and in particular, the coreathetotid subtype. Coreathetotid children are characterized by low muscular tone, involuntary movements and loss of postural stability. For these reasons, it would be interesting to understand if the TS could promote adaptive changes in the gait patterns of children with a clinical diagnosis totally different from children with spastic CP. It would also be important to analyze the immediate effects of Therasuit on orthostatic postural control (and to calculate the symmetry index) to determine its potential in promoting a more symmetric postural alignment, a fundamental condition for all functional motor tasks. Finally, studies addressing the immediate effects of Therasuit on gait kinematic parameters in children with US-CP using other methodologies, such as muscle recruitment data, extracted from electromyography, could allow a better identification of the neuromuscular behavior, thereby providing better information and guidance for the placements of the Therasuit elastic cords.

## Conclusion

The results in this study should be analyzed with caution due to the limitations previously mentioned (e.g., small convenience sample with minimal spasticity degree). Despite this, our results seem to suggest that wearing a Therasuit promotes: (1) positive kinematic changes on gait pattern in the paretic lower limb; (2) decrease hip flexion angles at initial contact in both lower limbs; (3) increase of the extension pattern at the hip joint during stance phase in the paretic lower limb, and a decrease of the flexion pattern during swing phase in both lower limbs; (4) a decrease of the equinus-foot pattern at the ankle joint in the paretic lower limb, during whole gait cycle, particularly on TS condition (with elastics). As this is the first study addressing the immediate effects of Therasuit on the gait pattern of children with hemiplegic CP, these findings may have important clinical implications.

Although our research only focused on the immediate effects of wearing Therasuit, the results suggest that the TS may be included by physical therapists in gait training programs for children with CP as an alternative dynamic orthosis, which may drive the child to use a new motor program, resulting in a safer and more functional gait pattern. Thus, our findings add to the growing body of evidence supporting the efficacy of Therasuit therapy for treating gait disorders in children with unilateral spastic CP.

## Data Availability

The raw data supporting the conclusions of this manuscript will be made available by the authors, without undue reservation, to any qualified researcher.

## Author Contributions

EM, RC, and RO conceived and designed the study. EM, JP, RC, and RO performed the experiments. EM, JV, and AD analyzed the data. EM, RC, RO, JP, AD, and JV wrote the paper.

### Conflict of Interest Statement

The authors declare that the research was conducted in the absence of any commercial or financial relationships that could be construed as a potential conflict of interest.
