# Supplementary material for: The Immediate Effects of a Dynamic Orthosis on Gait Patterns in Children With Unilateral Spastic Cerebral Palsy: A Kinematic Analysis
Source: Front Pediatr. 2019 Feb 21;7:42. doi: 10.3389/fped.2019.00042 (PMC6393373; doi:10.3389/fped.2019.00042)
Supplement: Supplementary file 1 [file Table_1.docx]

**Supplementary Table 1.** Passive ROM of the Lower Limbs Joints (degrees) in the paretic lower limb and non-paretic lower limbs (PLL/NPLL) of the participants.

| **Joint** | **Muscles** | **PARTICIPANTS** | | | | | | |
| --- | --- | --- | --- | --- | --- | --- | --- | --- |
|  |  | **1** | **2** | **3** | **4** | **5** | **6** | **7** |
| **HIP** | Extension | 10/10 | 5/10 | 10/10 | 0/15 | 5/10 | 10/15 | 15/15 |
|  | Hip flexion with knee flexed | 125/135 | 135/130 | 130/120 | 120/120 | 130/140 | 125/125 | 125/130 |
|  | Abduction with knee extension | 35/40 | 30/40 | 30/40 | 35/35 | 35/40 | 30/40 | 30/35 |
|  | Medial rotation | 50/55 | 60/60 | 50/50 | 60/55 | 50/50 | 55/55 | 35/35 |
|  | Lateral rotation | 40/50 | 40/50 | 45/50 | 50/55 | 30/35 | 35/45 | 35/40 |
| **KNEE** | Flexion | 140/150 | 140/150 | 130/140 | 150/150 | 145/150 | 140/150 | 135/140 |
|  | Extension | -5/0 | -5/0 | 0/0 | -5/0 | 0/0 | -5/0 | 0/0 |
| **ANKLE** | Dorsiflexion with knee extension | 10/25 | 5/20 | -10/10 | -10/15 | -10/15 | -10/10 | -5/15 |
|  | Plantar flexion | 50/50 | 50/50 | 50/50 | 35/35 | 35/40 | 30/50 | 40/50 |
|  | Inversion | 30/35 | 30/30 | 25/30 | 25/35 | 15/15 | 30/35 | 10/10 |
|  | Eversion | 15/15 | 10/20 | 20/20 | 20/25 | 5/10 | 10/15 | 25/35 |
