# Supplementary material for: The Immediate Effects of a Dynamic Orthosis on Gait Patterns in Children With Unilateral Spastic Cerebral Palsy: A Kinematic Analysis
Source: Front Pediatr. 2019 Feb 21;7:42. doi: 10.3389/fped.2019.00042 (PMC6393373; doi:10.3389/fped.2019.00042)
Supplement: Supplementary file 2 [file Table_2.docx]

**Supplementary Table 2.** Spasticity score in the paretic lower limb of the participants according to the Modified Ashworth Scale (MAS).

| **Joint** | **Muscles** | **PARTICIPANTS** | | | | | | |
| --- | --- | --- | --- | --- | --- | --- | --- | --- |
|  |  | **1** | **2** | **3** | **4** | **5** | **6** | **7** |
| **HIP** | Extensors | 0 | 0 | 0 | 0 | 0 | 0 | 0 |
|  | Flexors | 0 | 1 | 1 | 1 | 1 | 1+ | 0 |
|  | Abductors | 0 | 0 | 0 | 0 | 0 | 0 | 0 |
|  | Adductors | 0 | 1 | 0 | 0 | 0 | 1+ | 0 |
|  | Medial rotators | 0 | 1 | 0 | 0 | 0 | 1+ | 0 |
|  | Lateral rotators | 0 | 0 | 0 | 0 | 0 | 0 | 0 |
| **KNEE** | Flexors | 1 | 1 | 1 | 1 | 1 | 1+ | 1 |
|  | Extensors | 0 | 0 | 0 | 0 | 0 | 0 | 0 |
| **ANKLE/FOOT** | Dorsiflexors | 0 | 0 | 0 | 0 | 0 | 0 | 0 |
|  | Plantar flexors | 1 | 1 | 1+ | 1 | 1+ | 1+ | 1+ |
|  | Inversors | 0 | 1 | 1 | 1 | 1 | 1 | 1+ |
|  | Eversors | 1 | 0 | 0 | 0 | 0 | 0 | 0 |
