# Supplementary material for: The Immediate Effects of a Dynamic Orthosis on Gait Patterns in Children With Unilateral Spastic Cerebral Palsy: A Kinematic Analysis
Source: Front Pediatr. 2019 Feb 21;7:42. doi: 10.3389/fped.2019.00042 (PMC6393373; doi:10.3389/fped.2019.00042)
Supplement: Supplementary file 3 [file Table_3.docx]

**Supplementary Table 3.** Classification of the Gait Patterns Types of the participants according Winters, Gage & Hicks (1987).

| **Gait Type Classification** | **PARTICIPANTS** | | | | | | |
| --- | --- | --- | --- | --- | --- | --- | --- |
|  | **1** | **2** | **3** | **4** | **5** | **6** | **7** |
|  | Type I | Type II | Type II | Type II | Type II | Type IV | Type II |

**Note.** Type I is characterized by a “drop foot” which is noted most clearly in the swing phase of gait, due to inability to selectively control the ankle dorsi-flexors during this part of the gait cycle. More distal compromise than Types III e IV; Type II is characterized by true equinus in the stance phase of gait. To maintain the center of gravity on the foot, there is an increase in hip flexion and lumbar lordosis; Type III is characterized by impaired ankle dorsi-flexion in swing and a flexed, “stiff knee gait” as the result of hamstring/quadriceps co-contraction. Greater proximal compromise than types I and II. Type IV is characterized by much more marked proximal involvement than types I and II and the pattern is similar to that seen in spastic diplegia.
