# Supplementary material for: The Immediate Effects of a Dynamic Orthosis on Gait Patterns in Children With Unilateral Spastic Cerebral Palsy: A Kinematic Analysis
Source: Front Pediatr. 2019 Feb 21;7:42. doi: 10.3389/fped.2019.00042 (PMC6393373; doi:10.3389/fped.2019.00042)
Supplement: Supplementary file 4 [file Table_4.docx]

**Supplementary Table 4.** Decision making for the placement of the elastic cords used in each of the seven children.

| **Body Segments** | **Muscles** | **PARTICIPANTS** | | | | | | | **GOALS** | **Elastics cords**  **Positions** |
| --- | --- | --- | --- | --- | --- | --- | --- | --- | --- | --- |
|  |  | **1** | **2** | **3** | **4** | **5** | **6** | **7** |  |  |
| **SCAPULAR GIRDLE and TRUNK** | Scapular adductors  Vest elastic cords | NO | NO | NO | NO | YES | NO | NO | To support scapular region and promote rhomboid action (more trunk extension pattern) |   **POSTERIOR VIEW**  Bilateral |
|  | Scapular adductors  Vest elastic cords | YES | YES | YES | YES | NO | YES | NO | To support scapular region and promote rhomboid action and correct anteversion of shoulders (more trunk extension pattern) |   **POSTERIOR VIEW**  **Bilateral** |
|  | Abdominal muscles  Front connections between shorts and vest (bilateral) | YES | YES | YES | YES | YES | YES | YES | To promote Abdominal support and better co-contraction between trunk flexors and extensors |   **FRONT VIEW**  **Bilateral** |
|  | Trunk Extensors/ stabilizers  Connections between shorts and vest (bilateral) | YES | YES | YES | YES | YES | YES | YES | To support and promote trunk extensors and better co-contraction between trunk flexors and extensors |   **POSTERIOR VIEW**  **Bilateral** |
|  | Trunk Lateral flexors  (Abdominal obliques)  Lateral connections between shorts and vest | NO | NO | NO | NO | YES | NO | YES | To minimize the lateral flexion of the trunk on the non-paretic side  and promote better trunk alignment |   **LATERAL VIEW**  **Paretic side** |
|  | Trunk Lateral flexors  (Abdominal obliques)  Lateral connections between shorts and vest | YES | YES | YES | YES | NO | YES | NO | To minimize the lateral flexion of the trunk on the non-paretic side  and promote better trunk alignment | **LATERAL VIEW**  **Paretic side** |

| **Body Segments** | **Muscles** | **PARTICIPANTS** | | | | | | | **GOALS** | **Elastics cords**  **Positions** |
| --- | --- | --- | --- | --- | --- | --- | --- | --- | --- | --- |
|  |  | **1** | **2** | **3** | **4** | **5** | **6** | **7** |  |  |
| **HIP and PÉLVIS** | Hip Extensors (gluteus maximum muscles)  Elastics bands on shorts | YES | YES | YES | YES | YES | YES | YES | To support gluteus group and promote more hip extension pattern and consequently to minimize the hip flexion pattern |   **POSTERIOR VIEW**  **Bilateral** |
|  | Gluteus Elastics bands on shorts | YES | YES | YES | YES | YES | YES | YES | Improve lateral paretic pelvis stabilization during the stance phase and promote more lateral rotation on the Paretic Lower Limb |   **LATERAL VIEW**  **Paretic Lower Limb** |
|  | Lateral rotators  Connections between shorts and knee pads | YES | YES | YES | NO | NO | NO | NO | To promote more hip external rotation and minimize hip internal rotation |   **FRONT VIEW**  **Paretic Lower Limb** |
|  | Lateral rotators  Connections between shorts and knee pads | NO | NO | NO | NO | NO | YES | NO | To promote more hip external rotation and minimize hip internal rotation |   **LATERAL VIEW**  **Paretic Lower Limb** |
| **KNEE** | Knee Extensors  Front Connections between shorts and knee pads | YES | YES | YES | YES | YES | YES | YES | Promote support for quadriceps muscle, rectus femoral function and to minimize the knee extensors weakness and flexion pattern |   **FRONT VIEW**  **Paretic Lower limb** |
|  | Hip Flexors  Connections between shorts and knee pads | NO | YES | NO | NO | YES | NO | YES | To support for knee flexors and correct the knee hyperextension | **POSTERIOR VIEW**  **Paretic Lower Limb** |

| **Body Segments** | **Muscles** | **PARTICIPANTS** | | | | | | | **GOALS** | **Elastics cords**  **Positions** |
| --- | --- | --- | --- | --- | --- | --- | --- | --- | --- | --- |
|  |  | **1** | **2** | **3** | **4** | **5** | **6** | **7** |  |  |
| **TIBIA** | Connection between knee pads and shoes | NO | YES | NO | YES | NO | YES | NO | To correct internal tibial rotation |   **LATERAL VIEW**  **Paretic Lower Limb** |
|  | Connection between knee pads and shoes | NO | NO | YES | NO | YES | NO | YES | To correct external tibial rotation |     **LATERAL VIEW**  **Paretic Lower Limb** |
| **ANKLE and FOOT** | Dorsiflexors  Connections between knee pads and shoes | YES | YES | YES | YES | YES | YES | YES | Promote more dorsiflexion during stance phase and correct equinus foot | **FRONT VIEW**  **Paretic Lower Limb** |
|  | Connections between knee pads and shoes | NO | YES | NO | NO | YES | YES | NO | To correct foot pronation |   **LATERAL VIEW**  **Paretic Lower Limb** |
|  | Connections between knee pads and shoes | NO | NO | NO | YES | NO | NO | YES | To correct foot supination |   **LATERAL VIEW**  **Paretic Lower Limb** |

**Note.** The placement of the elastics was also based on the following general principles: i) Promote a better central stability or a greater activation of the stabilizing muscles of the trunk; ii) Promote a greater proprioceptive load in the paretic lower limb; iii) Promote a greater activation of the extensor muscles of the hip (maximum gluteus) and of the knee (quadriceps) and of the dorsal flexors at the ankle, in the paretic lower limb. The tension of the elastic cords was applied according to each child’s individual response.
