# Supplementary material for: The Immediate Effects of a Dynamic Orthosis on Gait Patterns in Children With Unilateral Spastic Cerebral Palsy: A Kinematic Analysis
Source: Front Pediatr. 2019 Feb 21;7:42. doi: 10.3389/fped.2019.00042 (PMC6393373; doi:10.3389/fped.2019.00042)
Supplement: Supplementary file 5 [file Image_1.pdf]

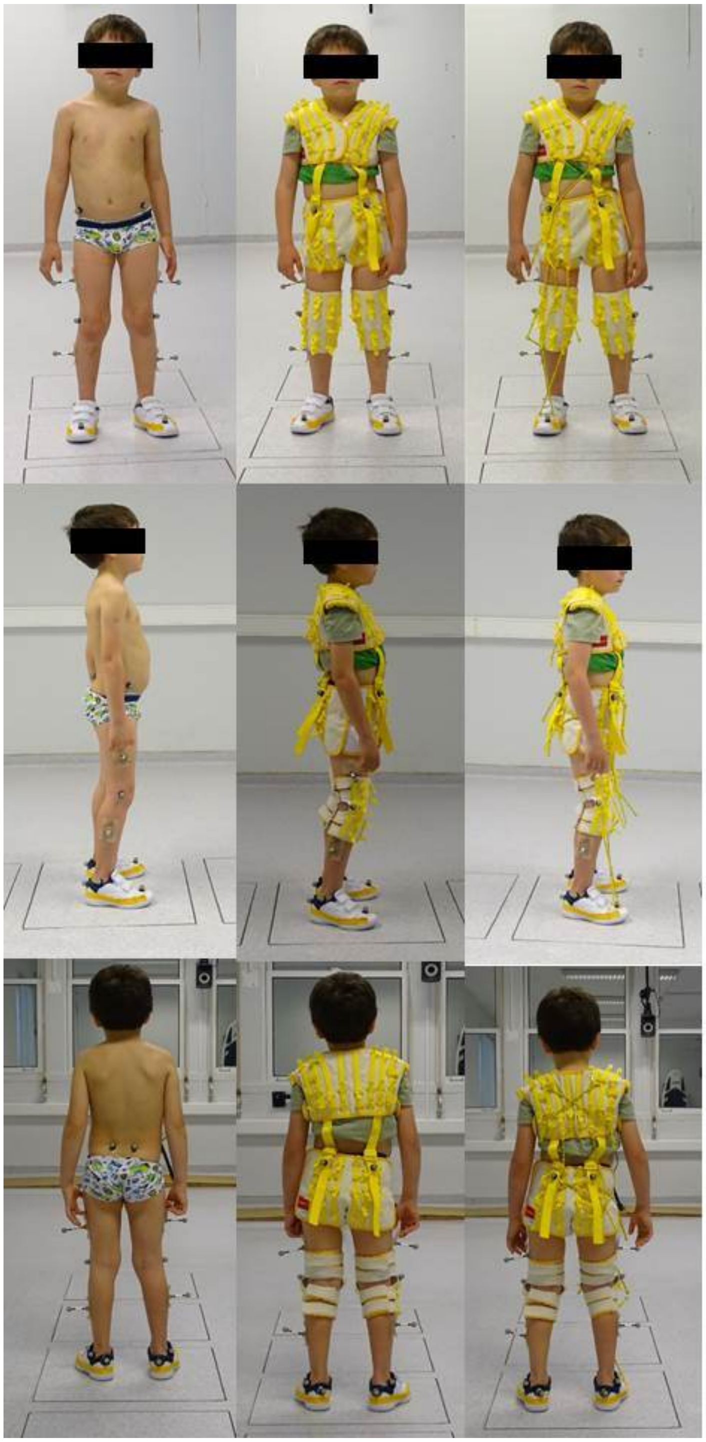

Supplementary Figure 1. Placement of the markers in the three conditions: Baseline (left); Therasuit without elastics (center); Therasuit (right).
